# Surfactant-dependant thermally induced nonlinear optical properties of l-ascorbic acid-stabilized colloidal GNPs and GNP–PVP thin films

**DOI:** 10.1039/c9ra01598a

**Published:** 2019-05-17

**Authors:** A. L. Sunatkari, S. S. Talwatkar, Y. S. Tamgadge, G. G. Muley

**Affiliations:** Department of Physics, Siddhartha College of Arts, Science and Commerce Fort Mumbai-400001 India ashok.sunatkari@rediffmail.com; Department of Physics, N. G. Aacharya and D. K. Maratha College of Arts, Science and Commerce Chembur Mumbai-400071 India; Department of Physics, Mahatma Phule Arts, Commerce & S. C. Science College Warud-444906 India; Department of Physics, Sant Gadge Baba Amravati University Amravati-444602 India

## Abstract

Gold nanoparticle (GNP) colloids stabilized with various concentrations of l-ascorbic acid were synthesized by the chemical reduction method and characterized by UV-Vis spectroscopy, XRD, FT-IR spectroscopy and TEM. XRD and TEM studies confirmed the reduction in particle size with the stabilizer concentration. UV-Vis spectra showed a blue shift in the LSPR peak. FT-IR peaks ascertained the strong encapsulation of GNPs with l-ascorbic acid functional groups. The nonlinear optical (NLO) properties of colloidal GNPs and GNP–PVP composite thin film were investigated using the Z-scan technique with CW laser excitation at 632.8 nm. The effects of stabilizer concentrations on nonlinear refractive index (*n*_2_), nonlinear absorption coefficient (*β*) and third-order susceptibility (*χ*^(3)^) of colloidal GNPs and GNP–PVP composite thin films were investigated. The values of the NLO parameters for the thin films were as large as *n*_2_ = 10^−5^ cm^2^ W^−1^, *β* = 10^−5^ cm W^−1^ and *χ*^(3)^_eff_ = 10^−5^ esu. For colloidal GNPs, these parameters were *n*_2_ = 10^−6^ cm^2^ W^−1^, *β* = 10^−6^ cm W^−1^ and *χ*^(3)^_eff_ = 10^−7^ esu. In both these cases, the NLO parameter values were found to decrease as the stabilizer concentration increased from 1 to 5 mM. The considerable enhancement in the NLO parameters may be attributed to the thermal lensing effect originating from the thermo-optic phenomenon. From the results, the influence of the concentration of the stabilizer on the NLO properties is obvious.

## Abstract 2

Gold nanoparticle (GNP) colloids stabilized with various concentrations of l-ascorbic acid were synthesized by the chemical reduction method and characterized by UV-Vis spectroscopy, XRD, FT-IR spectroscopy and TEM.

## Graphical abstract

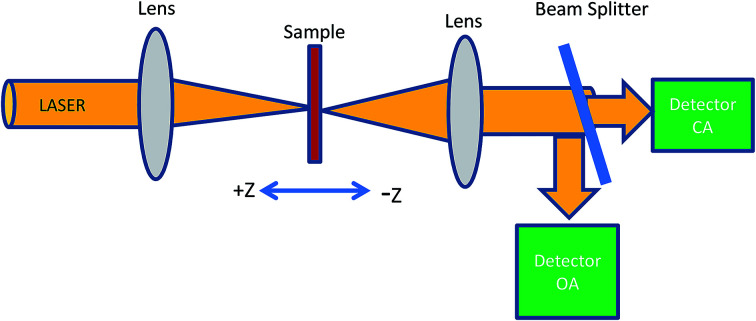

## Introduction

Nanocomposite materials, owing to their enormous novel applications in photonic devices such as optical switching, information storage, computing, signal processing, and optical limiting and imaging,^1–6^ have attracted widespread scientific and industrial attention in the last several years. Recent advances in polymer science have validated the capability to realize nanoparticle-embedded polymer composite materials exhibiting designed thermal, mechanical, linear and nonlinear optical properties.^7–12^ The noble metal-polymer composite thin film is one of the interesting optoelectronic materials due to its strong localized surface plasmon resonance (LSPR) absorption in metallic nanoparticles and large enhancement in third-order nonlinear optical (NLO) properties. In addition, the advantages associated with polymers such as a high damage threshold, simplicity of realization into photonic devices and low processing costs make them excellent hosts for nanoparticles.^13^

To achieve large optical nonlinearity and special optical absorption, organic/inorganic materials, noble metal nanoparticles, and carbon-based materials are embedded into various host dielectrics and polymer nanocomposites.^14,15^ Liao *et al.*^16,17^ and Fukumi *et al.*^18^ obtained third-order NLO susceptibility as large as 10^−6^ to 10^−7^ esu for the composite films containing gold nanoparticles embedded in an oxide matrix such as Au–SiO_2_ and Au–TiO_2_. In this study, they established the dependence of *χ*^(3)^ on the size of the gold nanoparticles and concentration. Fang *et al.*^19^ reported the third-order optical nonlinearities of gold nanoparticles incorporated in mesoporous silica thin films. In this study, they found that the linear and nonlinear optical properties can be tuned by changing the factors such as particle size, shape, and filling factor as well as by changing experimental conditions such as the use of different surfactants and calcination. Nanoparticles have a high specific surface area and their high surface energy must be stabilized to prevent further agglomeration. This can be achieved by wisely selecting suitable surfactants with moieties that introduce steric repulsion between them.

To date, a common choice for a metal nanoparticle (MNP) surfactant is the thiol-mediated binding of ligands.^20^ Aniline, long-chain amines, and carboxylic compounds have been used as stabilizers for the synthesis of MNPs.^21^ Researchers have also studied the roles of polyvinylpyrrolidone (PVP), polyacrylate and polyacrylamide as protective agents, which can effectively alter the shape, size, stability and linear optical properties of MNPs.^22^ More recently, researchers have diverted their attention to the binding of metal NPs with amino acids.^23–25^ Amino acids are inherently compatible and one of the common amino acid is l-ascorbic acid, which has a zwitterionic structure. On functionalization of MNPs with l-ascorbic acid molecules, they can easily facilitate the interaction and hence have a potential to bring drastic changes in NLO properties. Joshi *et al.*^26^ reported the synthesis of l-lysine-capped gold nanoparticles. Bhargava *et al.*^27^ reported the synthesis of gold nanoparticles using amino acids such as l-tyrosine and glycyl-l-tyrosine as reducing agents. Furthermore, reports on the preparation of various metal nanoparticles through a green method are available in the literature, in which l-ascorbic acid is used as a reducing agent.^27–33^ To the best of our knowledge, reports are not available on the use of l-ascorbic acid as a surfactant in the synthesis of gold nanoparticles (GNPs), and the effect of its concentration on linear optical and NLO properties has not been comprehensively explored.

In the present study, we aim to synthesize GNPs in the colloidal form with various concentrations of the surfactant l-ascorbic acid, followed by their dispersion into the PVP matrix to fabricate thin films by a spin-coating method. The effects of l-ascorbic acid concentration on LSPR, size of GNPs, nonlinear refractive index (*n*_2_), nonlinear absorption coefficient (*β*), and third-order susceptibility (*χ*^(3)^) of colloidal GNPs as well as GNP–PVP composite thin films were investigated under CW laser excitation (632.8 nm) using the well-known Z-scan technique. A comparative analysis of NLO parameter values of colloidal GNPs and GNP–PVP composites was conducted. The mechanism responsible for the considerable enhancement in NLO parameters was presented.

## Chemicals and experimental procedure

### Materials

Gold(iii) chloride hydrate (HAuCl_4_, 99.999% purity), sodium borohydride (NaBH_4_, 98% purity), l-ascorbic acid (99% purity) and PVP (MW 10 000) were purchased from Sigma-Aldrich and used without further purification. Double distilled water was used in the synthesis process. l-Ascorbic acid was used as a capping agent and NaBH_4_ was used as a reducing agent.

### Synthesis of l-ascorbic acid-protected colloidal GNP suspension

GNPs stabilized in various concentrations of stabilizer (l-ascorbic acid) were synthesized by the chemical reduction method. Stock solutions of HAuCl_4_ (5 mM) and NaBH_4_ (10 mM) were prepared. l-Ascorbic acid solutions with 1, 2.5, and 5 mM concentrations were prepared by dissolving an appropriate amount in distilled water. All solutions were kept in an ice bath for 20 min. In a 200 ml volumetric flask, 30 ml distilled water, 10 ml NaBH_4_ and 20 mL l-ascorbic acid solutions were taken and stirred at 50 °C for 20 min to remove any excess NaBH_4_. GNP colloidal solution was obtained by dropwise addition of 20 ml gold precursor into the above mixture. The solution turned light pink (stable colour) in colour in 10 min, indicating the formation of GNPs. The as-synthesized GNP colloidal suspension was purified by repeated centrifugation and washing; it was stable for a long duration of 3 months.

### Fabrication of GNP–PVP nanocomposite thin films

PVP solutions (15 wt%) were prepared by dissolving 15 g PVP powder into 100 ml double distilled water and it was stirred for 1 h at 40 °C. An appropriate amount of gold colloidal solution stabilized in 1, 2.5 and 5 mM l-ascorbic acid was taken into three separate flasks and the PVP solution was mixed with continuous stirring for 1 h to get a homogeneous viscous mixture. This viscous mixture was ultrasonicated to obtain a stable suspension and used to fabricate thin films by the spin-coating method. The prepared thin films were used to study their NLO properties and linear optical properties.

### Nonlinear optical parameter measurements using Z-scan

**Fig. 1:**
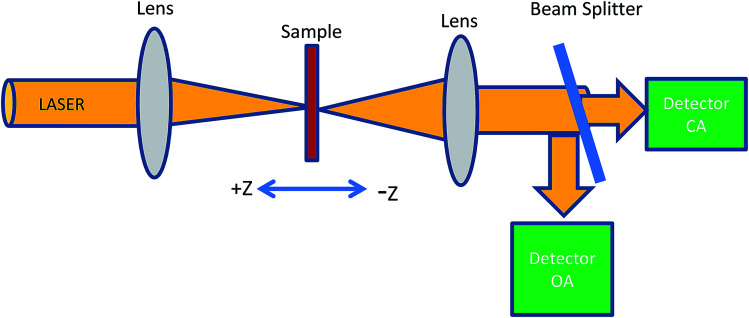
Schematic of a Z-scan set-up.

The Z-scan technique is the most reliable method for determining the nonlinear optical properties of materials such as nonlinear absorption and nonlinear refraction, which have been developed for a wide range of applications such as optical limiting, multi-photon polymerization and optical switching. The Z-scan technique is performed by translating a sample through the *Z*-axis (the beam waist) of a focused beam and then measuring the power transmitted through the sample. The two measurable quantities connected with the Z-scan are nonlinear absorption and nonlinear refraction. These parameters are associated with the imaginary and real parts of the third-order nonlinear susceptibility and provide vital information about the properties of the material. The experimental setup for single beam Z-scan technique is illustrated in Fig. 1.

The nonlinear absorption present in the sample can be estimated by the open aperture Z-scan technique. As the sample is translated through the focal region of the beam, the detector (OA) measures the total transmitted intensity. The normalized change in transmitted intensity can be approximated using the following equation:1
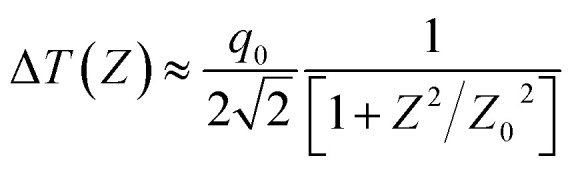


All the notations used here are as those given by Sheik-Bahae.^34,35^ If the beam experiences any nonlinear phase shift due to the sample as it is translated through the focal region, then, the fraction of light falling on the detector will vary due to the Kerr lens generated in the material by the intense laser beam. In this case, the signal measured by the detector will exhibit a peak and valley as the sample is translated through the *Z*-axis. The position of the peak and valley, relative to the *Z*-axis, depends on the sign of the nonlinear phase shift Δ*ϕ*_0_. If the phase shift is positive, self-focusing occurs and then, the peak will trail the valley. If the phase shift is negative, then, the valley will trail the peak and self-defocusing occurs. The magnitude of the phase shift can be determined from the change in the normalized transmittance between the peak and the valley, Δ*T*_PV_, using the following relation:2
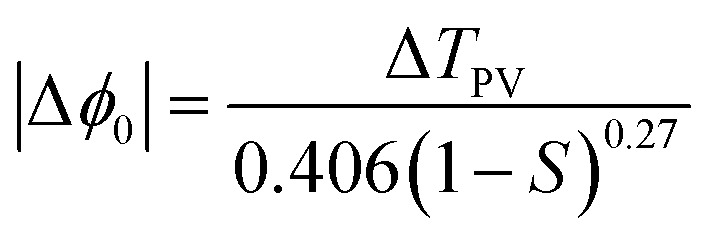
Here, *S* is the fraction of beam transmitted through the aperture. The nonlinear index, *n*_2_, can then be determined from the phase shift using the following equation:3
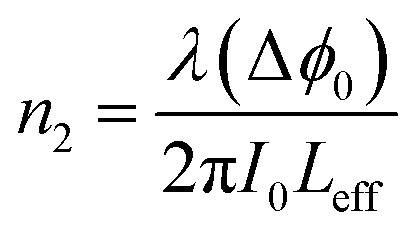


The third order nonlinear susceptibility can be obtained by the real and imaginary parts of third-order nonlinear optical susceptibility calculated from the estimated values of *n*_2_ (closed-aperture Z-scan) and *β* (open-aperture Z-scan) using the following relation:

4 *χ*^(3)^ = Re[*χ*^(3)^] + Im[*χ*^(3)^]

Here,5
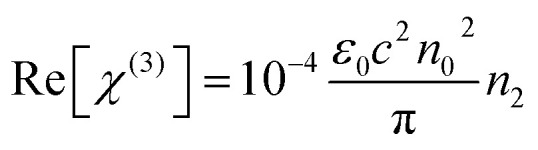
6
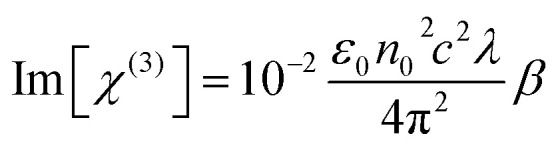


In this experiment, a low-power 10 mW He–Ne laser beam of wavelength 632 nm in the CW regime was used. Colloidal GNP samples were taken in the 1 mm path length quartz cuvette. In the present experimental set up, the laser beam was focussed using a lens of 20 cm focal length. The beam waist was 22 μm and Rayleigh length was *Z*_0_ = 2.4 mm. Thin film samples were mounted on the sample holder placed at the *Z*_0_ position and moved to and fro along the *Z*-axis. At each position on the *Z*-axis, the input beam energy and transmitted beam energy were measured with the help of the detector. The experiment was carried out for open aperture as well as closed aperture configuration to estimate the intensity-dependant nonlinear refraction and third-order nonlinear susceptibility of GNP colloids and GNP–PVP thin films. The sample experienced different intensities of laser light as it changed position along the focal plane, *i.e.*, the Gaussian beam experienced a nonlinear phase shift due to the translation of the sample through the focal region. Due to nonlinear refraction, the spatial beam broadening or narrowing occurred in the far field. In this case, the transmitted signal recorded by the detector exhibited a peak and valley as the sample moved along the focal region. The sign and the magnitude of nonlinear refraction could be deduced from the transmittance curve. The variation in transmitted intensity through the sample is provided in the Results section.

## Results and discussion

### UV-visible study of colloidal GNPs and GNP–PVP matrix

**Fig. 2:**
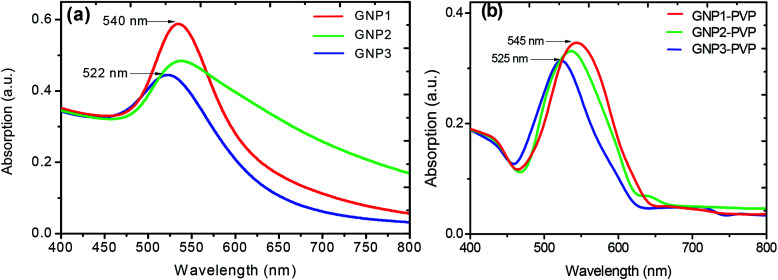
UV-visible spectra of l-ascorbic acid-stabilized (a) GNP colloidal suspension and (b) PVP–GNP thin films with 1, 2.5 and 5 mM concentrations of l-ascorbic acid.

The optical absorption spectra of l-ascorbic acid-stabilized colloidal GNP suspension and GNP–PVP thin film were recorded in the wavelength range from 200 to 800 nm at room temperature (Fig. 2a and b, respectively). Strong absorption peaks in the range of 540–522 nm can be observed in the visible region for the l-ascorbic acid-encapsulated GNP colloidal suspension; also, we can see strong absorption peaks at 545–525 nm for the GNP–PVP thin films, a signature property shown by spherical GNPs as proposed by the Mie theory, due to LSPR. The presence of a single LSPR peak suggests spherical shape of GNPs.^36–39^ It is noticed that when the stabilizer l-ascorbic acid concentration increased from 1 to 5 mM, the LSPR peak shifted to 520 nm. A blue shift in the LSPR peak from 540 to 522 nm for colloidal GNPs and from 545 to 525 nm for the GNP–PVP nanocomposite along with peak broadening indicated the decrease in GNP size as expected according to the Mie theory.

In Fig. 2b, the LSPR peak of GNP–PVP thin film indicates the incorporation of GNPs into the polymer matrix. A slight shift of 2–3 nm in the LSPR peak in the nanocomposite matrix compared to the colloidal suspension may be due to the change in the dielectric constant of the medium.^40–42^

The formation of smaller-sized gold NPs with the increase in the concentration of the stabilizer was also authenticated by TEM measurements. Alvarez *et al.*^43^ and Palpant *et al.*^44^ also reported a blue shift in LSPR on decreasing the particle size. They applied the time-dependant local density approximation (TDLDA) theory for calculations and found good agreement. They explained the effect as follows: the metal nanoparticle cluster has two regions: inner core and outer shell. The blue shift with the decrease in size is due to the screening effects caused by the polarisable inner medium, which can vanish at the outer shell of the particles. However, De *et al.*^45^ did not attribute the blue shift in LSPR to the size effect. They explained that the blue shift in LSPR is due to the electron transfer to the gold cluster from hydrogen in a reducing atmosphere, filling up its 5s band. The increase in s-electron density results in increase in Fermi energy and thus plasma frequency, which causes reduction in the absorption wavelength.

Interestingly, l-ascorbic acid-capped GNPs were stable for a long period without any aggregation, whereas free GNPs started to aggregate within two weeks at room temperature. Aryal *et al.*^46^ also noticed similar results when they stabilized gold nanoparticles by l-cysteine. Due to the surface potential reduction resulting from the transfer of electrons to GNPs from NaBH_4_, a reducing agent used in the synthesis process, nanoparticles started to aggregate.^47^ Guo *et al.*^48^ reported the aggregation of GNPs and attributed it to their different morphologies such as decahedrons, tetrahedrons, truncated tetrahedrons and cubes as GNPs have different chemical activities on different crystalline facets. Therefore, the strength of the binding force of the stabilizer l-ascorbic acid on different crystalline facets is not uniform. As a result, the stabilizer with a weak binding force on some facets will start desorbing and aggregating. Fig. 4e shows the TEM image of aggregated GNPs after three and a half months.

### Structural and morphological study of GNP–PVP composite thin films

**Fig. 3:**
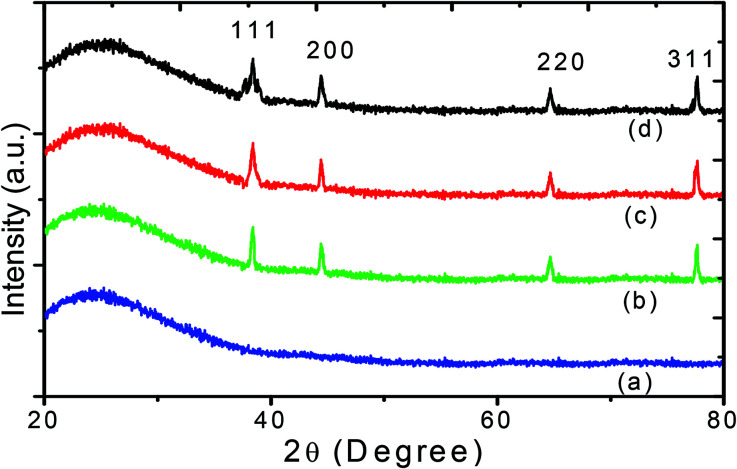
XRD patterns of (a) PVP and thin films implanted with GNPs stabilized with (b) 1, (c) 2.5 and (d) 5 mM l-ascorbic acid.

Fig. 3 shows the XRD patterns of nanocomposite thin films prepared without GNPs and with GNPs stabilized in various concentrations of l-ascorbic acid with four indexable peaks. The XRD analysis of PVP polymer and GNP–PVP nanocomposite thin films confirmed the presence and crystallinity of GNPs.

A very broad peak at around 24.35° is a characteristic peak of the amorphous polymer.^40^ Other diffraction peaks located at 38.50°, 44.42°, 64.67° and 77.65°, corresponding to the planes (111), (200), (220), and (311), belong to the face-centred-cubic-structured gold crystals and agree with the data given in the literature (JCPDS 01-1172).

**Fig. 4:**
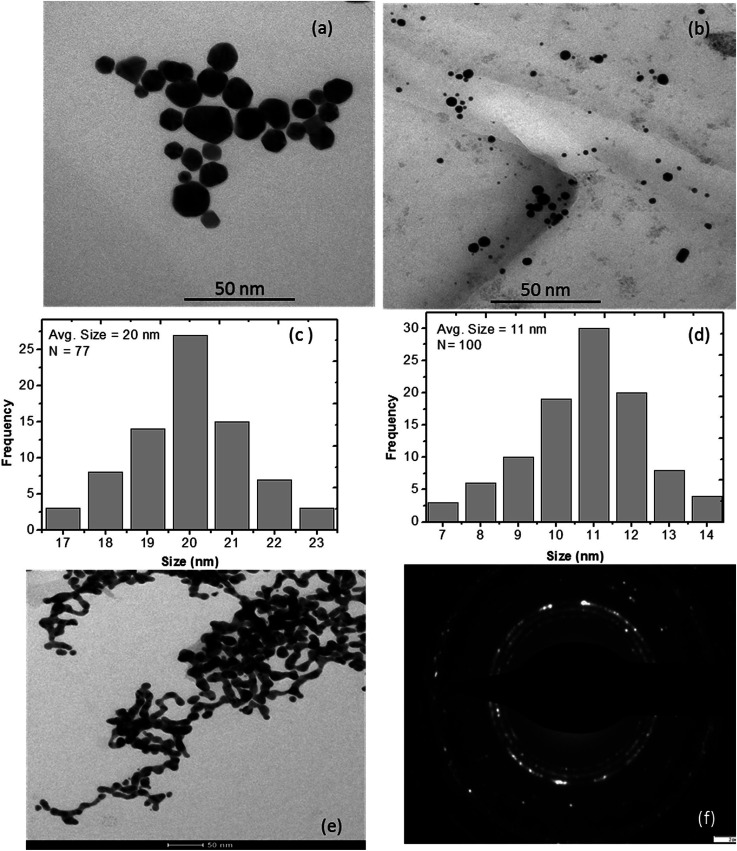
TEM images of aqueous colloidal suspension of GNPs stabilized with (a) 1, (b) 5 mM l-ascorbic acid; (c) and (d) show their histograms of particle size distribution, respectively; (e) aggregation of GNPs; (f) SAED pattern obtained for GNP colloidal suspension at a concentration of 5 mM l-ascorbic acid.

The particle sizes of GNPs were estimated at about 17, 14 and 9 nm and decreased with the increase in the stabilizer concentration. The TEM images shown in Fig. 4 also validate the decrease in the GNP size.

### TEM images

The more accurate sizes of GNPs can be obtained using TEM. TEM images of PVP thin film containing GNPs stabilized in 1, 2.5 and 5 mM l-ascorbic acid were obtained. Well-dispersed GNPs with almost uniform sizes were synthesized (Fig. 4a and b). Fig. 4c and d show their histograms of particle size distribution. The average sizes of GNPs were found to be 20, 16 and 11 nm for 1, 2.5 and 5 mM l-ascorbic acid concentrations, respectively. These results advocate the blue shift in the absorption spectra and decrease in the particle size. Fig. 4e shows the aggregation of GNPs after three and a half months. The selected area electron diffraction (SAED) pattern obtained for the GNP colloidal suspension at a concentration of 5 mM l-ascorbic acid is shown in Fig. 4f. The sizes obtained from XRD and TEM analyses are listed in Table 1.

**Table:** GNP sizes obtained from XRD and TEM analyses

Sample	l-Ascorbic acid concentration (mM)	Absorption wavelength (nm)	Size from XRD (nm)	Size from TEM (nm)
PVP–GNP1	1.0	545	17	20
PVP–GNP2	2.5	531	14	16
PVP–GNP3	5.0	525	9	11

### FTIR study of l-ascorbic acid and l-ascorbic acid-capped GNPs

**Fig. 5:**
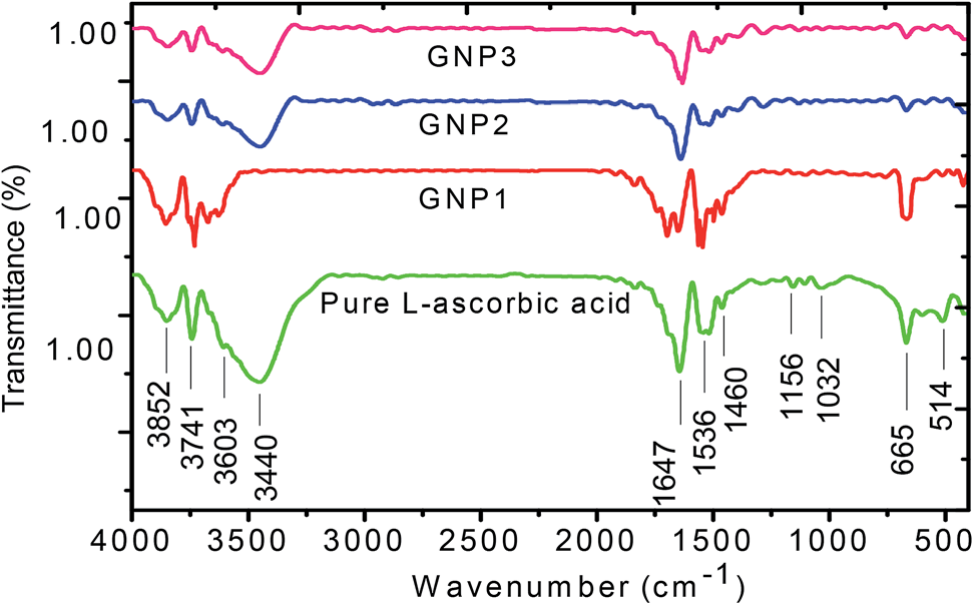
FT-IR spectra of pure l-ascorbic acid and GNPs stabilized with 1, 2.5 and 5 mM l-ascorbic acid.

FT-IR spectroscopy is a powerful tool to investigate how strongly GNPs are bonded to l-ascorbic acid. Fig. 5 shows the FT-IR spectra of pure l-ascorbic acid along with GNPs stabilized with various concentrations of l-ascorbic acid. The higher region of FT-IR spectra is known as the hydroxyl group region, *i.e.*, the O–H stretching region. The position and the intensity of peaks depend upon the strength of hydrogen bonding. All four bands above 3000 cm^−1^ are presumably associated with O–H stretching. The presence of functional groups C=O, C=C and C–H in l-ascorbic acid gives rise to bands around 1600–1750 cm^−1^. The vibrational bands appearing at 1536 cm^−1^ and 1460 cm^−1^ are due to the C=C stretching and C–H deformation. The peak in the spectra at 1101 cm^−1^ is mainly because of the stretching of the C–O–C group.^49,50^

The shift in the band position along with the decrease in the intensity for various concentrations of l-ascorbic acid suggests bonding with GNPs. As expected, the spectra show signature peaks from 1453 to 1652 cm^−1^ due to the stretching, bending and deformation of functional groups as a result of their attachment with the GNP surface. Various bands appearing in the spectra along with their tentative interactions are shown in detail in Table 2. The decrease in the intensity with concentration of the stabilizer l-ascorbic acid along with a significant shift in the peak position is a result of strong bonding between functional groups and GNPs.^51–53^

**Table:** Assignment of FT-IR peaks of l-ascorbic acid and GNPs stabilized in 1, 2.5 and 5 mM l-ascorbic acid^a^

Pure l-ascorbic acid (cm^−1^)	GNP1 (cm^−1^)	GNP2 (cm^−1^)	GNP3 (cm^−1^)	Assignment ref. 49–53
3852	3852	3853	3855	O–H str.
3741	3743	3745	3743	O–H str.
3603	—	—	—	O–H str.
3440	3444	3443	—	O–H str.
1694	1701	1695	1698	N–H bend.
1749	—	—	—	C=O str.
1647	1648	1649	1650	N–H def./C–C str.
1536	1547	1542	1551	N–H bend./C–O str.
1460	1453	1461	1464	CH_3_ asy. def./COO sym. str.
1156	—	1161	—	C–N/C–C–C sym. str.
1101	1098	—	—	C–N str.
1032	—	—	—	C–C str.
665	670	668	668	COO bend.
514	506	508	512	C–O def. Vib.

a Str. = stretching, bend. = bending, asy. = asymmetric, def. = deformation, Vib. = vibrational.

### Stability of GNPs

The stability of GNPs is a very vital factor in their applications. Here, l-ascorbic acid was used as a stabilizing agent. The aqueous solution of l-ascorbic acid-stabilized GNPs was centrifuged at 7000 rpm for 30 min. A very small amount of precipitate was seen at the bottom of the tube, which was soluble after completely shaking it. The colloidal suspension containing GNPs was kept under ambient conditions; no sign of aggregation was observed for up to 3 months. GNPs started to slowly aggregate, as seen in the TEM image shown in Fig. 4e, which was obtained on the 100^th^ day from synthesis. This demonstrates that l-ascorbic acid-stabilized GNPs are highly stable for around 3 months due to their excellent capping effect.

### NLO studies of colloidal GNPs and GNP–PVP nanocomposites

**Fig. 6:**
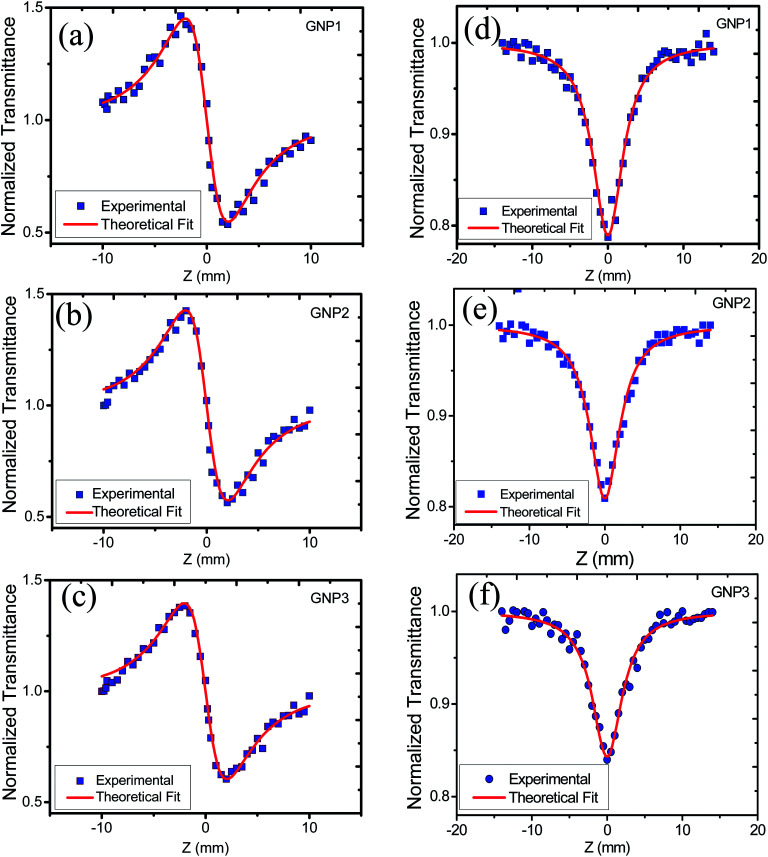
(a), (b) and (c) represent CA transmittance and (d), (e) and (f) represent OA transmittance of colloidal GNP suspensions stabilized in 1, 2.5 and 5 mM l-ascorbic acid, respectively.

**Fig. 7:**
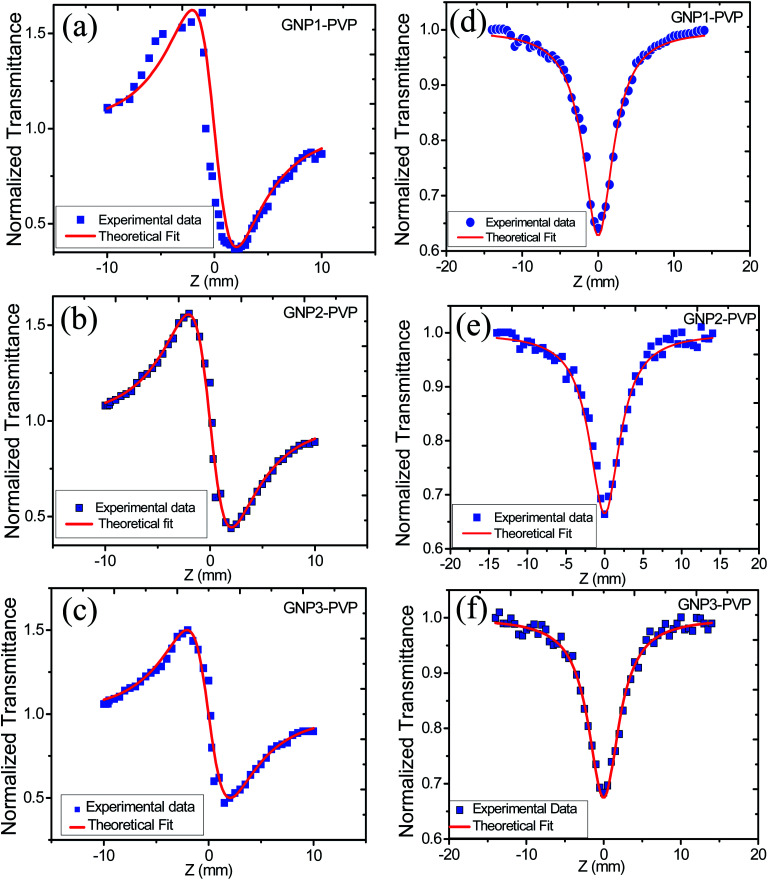
(a), (b) and (c) represent CA transmittance and (d), (e) and (f) represent OA transmittance of PVP thin films embedded with GNPs stabilized in 1, 2.5 and 5 mM l-ascorbic acid, respectively.

In order to study the effect of stabilizer concentration on the nonlinear refractive index (*n*_2_), nonlinear absorption coefficient (*β*), and third-order susceptibility (*χ*^(3)^_eff_) of colloidal GNPs and GNP–PVP composite thin films, the samples were investigated under CW laser excitation at a wavelength of 632.8 nm. Both closed aperture (CA) and open aperture (OA) Z-scans were performed. The normalized transmittance curves of GNP colloidal suspension and thin film are shown in Fig. 6 and 7, respectively.

CA data comprised both the effects of nonlinear refraction and absorption; hence, CA Z-scan data were divided with open aperture Z-scan data to exclude the effect of nonlinear absorption. The values of nonlinear refractive index, nonlinear absorption coefficient, and third-order nonlinear susceptibility were calculated and are provided in Table 3.

NLO parameters of aqueous suspension of GNPs and PVP–GNPs stabilized in 1, 2.5 and 5 mM concentrations of l-ascorbic acid

**Table:** 

Sample	GNP size (nm)	l-Ascorbic acid con (mM)	Δ*T*_P−V_	|Δ*ϕ*_0_|	*n*_2_ cm^2^ W^−1^ (10^−6^)	*β* cm W^−1^ (10^−6^)	Re *χ*^(3)^ esu (10^−7^)	Im *χ*^(3)^ esu (10^−11^)	*χ* ^(3)^_eff_ esu (10^−7^)
Colloidal GNPs									
GNP1	20	1.0	0.786	2.222	−1.43	3.38	5.23	2.40	5.23
GNP2	16	2.5	0.749	2.119	−1.35	4.11	4.95	2.92	4.95
GNP3	11	5.0	0.698	1.973	−1.27	4.54	4.63	3.22	4.63

**Table:** 

Sample	GNP Size (nm)	l-Ascorbic acid con. (mM)	Δ*T*_P−V_	|Δ*ϕ*_0_|	*n*_2_ cm^2^ W^−1^ (×10^−5^)	*β* cm W^−1^ (×10^−5^)	Re *χ*^(3)^ esu (×10^−5^)	Im *χ*^(3)^ esu (×10^−11^)	*χ* ^(3)^_eff_ esu (×10^−5^)
PVP–GNP thin films									
GNP1–PVP	20	1.0	1.085	3.06	−3.79	8.96	2.23	6.36	2.23
GNP2–PVP	16	2.5	0.968	2.73	−3.38	7.04	1.99	5.00	1.99
GNP3–PVP	11	5.0	0.871	2.46	−3.04	6.80	1.79	4.83	1.79

**Fig. 8:**
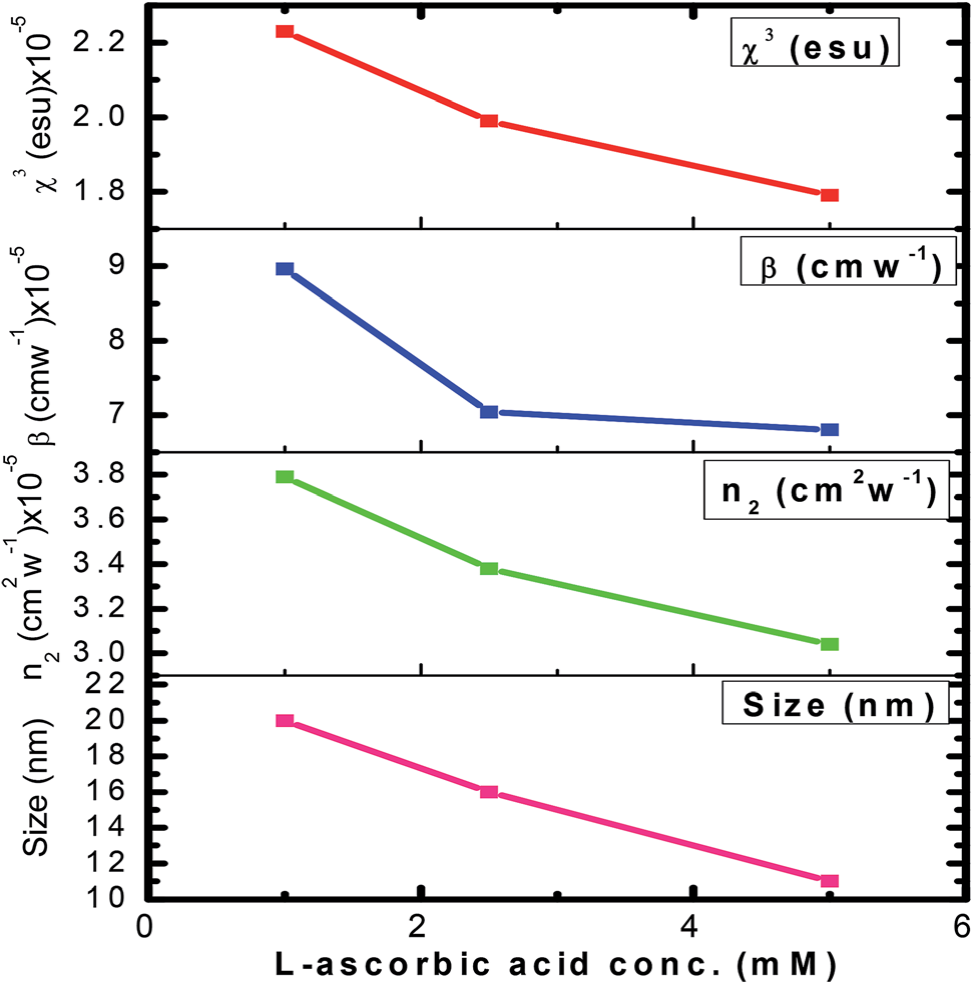
The variation of *n*_2_, *β* and *χ*^(3)^_eff_ with stabilizer l-ascorbic acid concentrations.

The variation in NLO parameters with the concentration of the stabilizer l-ascorbic acid is displayed in Fig. 8. Fig. 6 and 7, of CA transmittance curve, shows valley is trailing the peak indicating of self-defocusing nature of samples. Therefore, the sample showed a negative nonlinear refractive index. For colloidal GNPs, as the concentration of l-ascorbic acid increased, the nonlinear refractive index (*n*_2_) reduced from 1.43 × 10^−6^ cm^2^ W^−1^ to 1.27 × 10^−6^ cm^2^ W^−1^; for PVP–GNP thin films, it reduced from 3.79 × 10^−5^ cm^2^ W^−1^ to 3.04 × 10^−5^ cm^2^ W^−1^.

The effective third-order nonlinear susceptibility (*χ*^(3)^_eff_) value measured in colloidal GNP suspension varied from 5.23 × 10^−7^ to 4.63 × 10^−7^ esu, whereas in thin films, it varied from 2.23 × 10^−5^ to 1.79 × 10^−5^ esu. Furthermore, nonlinear absorption (*β*) was estimated in the order of 3.38–4.54 (×10^−6^ cm W^−1^) for colloidal GNPs, whereas in the GNP–PVP nanocomposites, it was in the order 8.96–6.90 (×10^−5^cm W^−1^). This suggests that the nonlinear refraction, third-order susceptibility and nonlinear absorption of GNPs in PVP thin films are better compared to the observations for the l-ascorbic acid-stabilized colloidal GNPs. Nonlinear refraction may comprise electronic and thermal contributions; however, in the CW (as we have used a CW laser) regime, thermal contributions dominate. This is also indicated by the Z-scan curves shown in Fig. 6 and 7 as Δ*T*_P−V_ is 1.7 times the Rayleigh range and nonlinearity is thermal in nature. Considerable enhancement in the nonlinear refractive index may be attributed to the thermally induced lensing effect in PVP–GNP thin films.^54,55^ The electrons with energy higher than the Fermi energy are called hot electrons. These hot electrons dissipate their excess energy through the scattering process. By doing so, the surrounding temperature increases and creates a temperature gradient; this causes a variation in the refractive index 
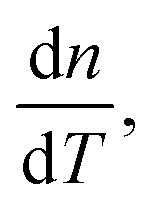
 which is called thermal lensing. Due to the thermal lensing effect, the phase shift *ϕ* experienced by the laser beam is 
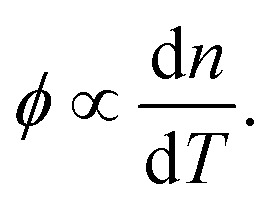
 In thin films, contributions from the end-phase curvature and thermal stress^56,57^ also play their roles. A larger expansion of the hotter centre of thin films compared to the cooler edge leads to end-face curvature contribution, and the cooler edge of thin films prevents the expansion of the hotter centre, thus producing thermal stress.

Furthermore, Dolgaleva *et al.*^58^ found ten-fold enhancement in NLO responses and attributed this to the local field effect that originated due to hot spots (electrons). They explained that in nanocomposites, surface plasmon excitations are confined to a small nano-region called as hot spots. The electromagnetic energy stored in these hot spots associated with localised plasmons leads to a local field that can exceed the intensity of the applied field by four or five times in magnitude. The high local fields in the hot spots result in considerable enhancement in NLO responses. The local field factor also becomes dependant on the dielectric constant (*ε*_d_) of the dielectric host matrix as the local field factor (*f*) takes the form of 
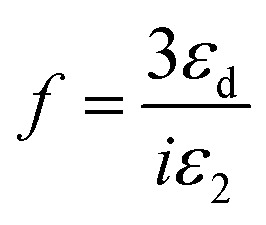
 near SPR. Therefore, the matrix with a larger dielectric constant makes the composite gain a larger value of *f* and the SPR peak also shifts due to the energy dependence of the dielectric constant. Hence, a metal particle-embedded composite matrix usually has a larger optical nonlinear response near the individual SPR as noticed in the present case. Specifically, third-order nonlinear optical susceptibility (*χ*^(3)^) becomes proportional to the fourth power of the local field factor under the condition of low metal concentration.^59–63^

Finally, considerable enhancement in the NLO responses of the PVP–GNP matrix may be due to LSPR, thermo-optic effect, thermal lensing effect, and local field factor. However, detailed analysis may be required to completely comprehend the exact underlying phenomenon contributing to the high value of nonlinear parameters shown by the PVP–GNP matrix. It is also observed from Table 3 that as the stabilizer concentration increases, the size of GNPs, nonlinear refractive index and third-order susceptibility decrease. Size-dependant nonlinearity was also noticed by Shahriari *et al.*^61^ and Fukumi *et al.*^18^ They established the relation that *χ*^(3)^ is proportional to the fourth power of the radius of nanocrystals, volume fraction of metal colloid particles and *χ*^(3)^_m_ of metal particles. We have compared the estimated NLO parameter values with the reported values in the literature. Our samples showed enhanced values of the NLO parameters. Table 4 shows the reported values of the NLO parameters of colloidal GNPs and GNP–PVP matrix.

**Table:** Comparison of the measured values of NLO parameters with the values reported in the literature

Sample	Wavelength (nm)	*n*_2_ (cm^2^ W^−1^)	*β* (cm W^−1^)	*χ* ^(3)^ (esu)	Ref.
Colloidal AgNPs	632 (CW)	−4.04 × 10^−7^	—	—	62
GNP–Al_2_O_3_ matrix	532 (CW)	7.62 × 10^−9^	−1.31 × 10^−3^	6.25 × 10^−8^	64
GNP–SiO_2_ matrix	532 (CW)	2.97 × 10^−9^	−0.12 × 10^−3^	2.7 × 10^−8^	64
Colloidal GNPs	632 (CW)	−2.6 × 10^−8^	—	—	39
GNP–ZnO matrix	532 (CW)	−1.31 × 10^−9^	−0.08 × 10^−3^	2.46 × 10^−8^	64
Colloidal GNPs	514 (CW)	−2.68 × 10^−6^	—	—	65
Colloidal GNPs	633 (CW)	−2.0 × 10^−7^	—		21
GNP–Eu_2_O_3_	633 (CW)	1.1 × 10^−8^	0.54	2.8 × 10^−3^	66
Colloidal GNPs	532 (CW)	0.31 × 10^−7^	—	—	67
Colloidal GNPs	632 (CW)	−(1.43–1.27) × 10^−6^	(3.38–4.54) × 10^−6^	(5.23–4.63) × 10^−7^	This work
GNP–PVP	632 (CW)	−(3.79–3.04) × 10^−5^	(8.96–6.80) × 10^−5^	(2.23–1.79) × 10^−6^	This work

## Conclusion

In summary, GNPs stabilized with various concentrations of l-ascorbic acid as the stabilizer were synthesized by the chemical reduction method and then characterized. UV-Vis spectroscopy proved the formation of spherical GNPs by indicating a strong blue shift in the localized surface plasma resonance (LSPR) peak from 545 to 522 nm for the GNP colloids and from 545 to 525 nm for the PVP–GNP composite thin film. The strong bonding between the functional groups of l-ascorbic acid and GNPs was confirmed from the FT-IR study. The effect of the concentration of stabilizer on the size of GNPs was substantiated from XRD and TEM studies. It was found that the size of GNPs decreased as we increased the concentration of the stabilizer. The nonlinear optical properties of GNP colloids and PVP–GNP composite thin films were studied by the Z-scan technique in the CW regime. A considerable enhancement in optical nonlinearity was noticed for both colloidal GNPs and PVP–GNP composite matrix owing to the thermal lensing effect originating from the thermo-optic phenomenon. The calculated values of nonlinear refractive index (*n*_2_), nonlinear absorption coefficient (*β*) and third-order susceptibility (*χ*^(3)^_eff_) of the colloidal suspension of GNPs were found in the order of 1.43–1.27 (×10^−6^ cm^2^ W^−1^), 3.38–4.54 (×10^−6^ cm W^−1^) and 5.23–4.63 (×10^−7^ esu), respectively. Furthermore, ten-fold enhancement in the nonlinear refractive index *n*_2_ = 10^−5^ cm^2^ W^−1^ and 100-fold enhancement in *χ*^(3)^_eff_ = 10^−5^ esu for the PVP–GNP composite matrix were also noticed. Moreover, the NLO parameters decreased as the stabilizer concentration in the colloidal suspension as well as in the PVP–GNP matrix increased. Based on these experimental findings, the synthesized thin films are claimed as promising materials for optical limiters, sensors and other applications of optoelectronic devices.

## Conflicts of interest

There are no conflicts to declare.

## Supplementary Material

## References

[cit1] Zheng Q., He G. S., Prasad P. N. (2009). Chem. Phys. Lett..

[cit2] Makarov N., Rebane A., Drobizhev M., Wolleb H., Spahni H. (2007). J. Opt. Soc. Am. B.

[cit3] Huang T., Hao Z., Gong H., Liu Z., Xiao S., Li S., Zhai Y., You S., Wang Q., Qin J. (2008). Chem. Phys. Lett..

[cit4] Liu M. O., Tai C. H., Hu A. T., Wei T. H. (2004). J. Organomet. Chem..

[cit5] Henari F. Z. (2001). J. Opt. A: Pure Appl. Opt..

[cit6] Ishikawa H. , Ultrafast all-optical signal processing devices, Wiley, U. K., 2008

[cit7] Gu J., Yan Y., Zhao Y. S., Yao J. (2012). Adv. Mater..

[cit8] Ajayan P. M. , Schadler L. S. and Braun P. V., Nanocomposite Science and Technology, Wiley VCH Verlag, Weinheim, 2003

[cit9] Morimune S., Kotera M., Nishino T., Goto K., Hata K. (2011). Macromolecules.

[cit10] Hou Y., Tang J., Zhang H., Qian C., Feng Y., Liu J. (2009). ACS Nano.

[cit11] Senge M., Fazekas M., Notaras E., Blau W., Zawadzka M., Locos O., Mhuircheartaigh E. N. (2007). Adv. Mater..

[cit12] Ishchenko A. A. (2008). Pure Appl. Chem..

[cit13] Sreeja S., Sreedhanya S., Smijesh N., Philip R., Muneera C. I. (2013). J. Mater. Chem. C.

[cit14] Gu J., Yan Y., Zhao Y. S., Yao J. (2012). Adv. Mater..

[cit15] Hou Y., Tang J., Zhang H., Qian C., Feng Y., Liu J. (2009). ACS Nano.

[cit16] Liao H. B., Xiao R. F., Fu J. S., Yu P., Wong G. K. L., Sheng P. (1997). Appl. Phys. Lett..

[cit17] Liao H. B., Xiao R. F., Wang H., Wong K. S., Wong G. K. L. (1998). Appl. Phys. Lett..

[cit18] Fukumi K., Chayahara A., Kadono K., Sakaguchi T., Horino Y., Miya M., Fuji K., Hayakawa J., Satou M. (1994). J. Appl. Phys..

[cit19] Fang J., Qin S., Zhang X., Nie Y., Wang F. (2012). RSC Adv..

[cit20] He S. T., Yao J. N., Xie S. S., Gao H. J., Pang S. J. (2001). J. Phys. D: Appl. Phys..

[cit21] Hiramatsu H., Osterloh F. E. (2004). Chem. Mater..

[cit22] Chen M., Wang L., Han J., Zhang J., Li Z., Qian D. (2006). J. Phys. Chem. B.

[cit23] Selvakannan P. R., Mandal S., Phadtare S., Pasricha R., Sastry M. (2003). Langmuir.

[cit24] Talwatkar S. S., Sunatkari A. L., Tamgadge Y. S., Pahurkar V. G., Muley G. G. (2015). J. Nanostruct. Chem..

[cit25] Talwatkar S. S., Tamgadge Y. S., Sunatkari A. L., Gambhire A. B., Muley G. G. (2014). Solid State Sci..

[cit26] Joshi H., Shirude P. S., Bansal V., Ganesh K. N., Sastry M. (2004). J. Phys. Chem. B.

[cit27] Bhargava S. K., Booth J. M., Agrawal S., Coloe P., Kar G. (2005). Langmuir.

[cit28] Xiong J., Wang Y., Xue Q., Wu X. (2011). Green Chem..

[cit29] Ryu H. R., Sanchez L., Keul H. A., Raj A., Bockstaller M. R. (2008). Angew. Chem., Int. Ed..

[cit30] Obliosca J. M., Harvey I., Arellano J., Huang M. H., Arco S. D. (2010). Mater. Lett..

[cit31] Malassisa L., Dreyfusb R., Murphy R. J., Hough L. A., Donnio B., Murraya C. (2016). RSC Adv..

[cit32] Bastus N. G., Comenge J., Puntes V. (2011). Langmuir.

[cit33] Vemula P., Aslam U., AjayMallia V., John G. (2007). Chem. Mater..

[cit34] Sheik-Bahae M., Said A., Wei T., Hagan D., Van Stryland E. (1990). IEEE J. Quantum Electron..

[cit35] Sheik-Bahae M., Said A., Van Stryland E. (1989). Opt. Lett..

[cit36] Tamgadge Y. S., Talwatkar S. S., Sunatkari A. L., Pahurkar V. G., Muley G. G. (2015). Thin solid films.

[cit37] Tamgadge Y. S., Sunatkari A. L., Talwatkar S. S., Pahurkar V. G., Muley G. G. (2016). Opt. Mater..

[cit38] Tamgadge Y. S., Pahurkar V. G., Talwatkar S. S., Sunatkari A. L., Muley G. G. (2015). Appl. Phys..

[cit39] Kelly K. L., Coronado E., Zhao L. L., Schatz G. C. (2003). J. Phys. Chem. B.

[cit40] Chen S., Kimura K. (1999). Langmuir.

[cit41] Sau T. K., Rogach A. L., Jackel F., Klar T. A., Feldmann J. (2010). Adv. Mater..

[cit42] Boyd R. W., Gehr R. J., Fischer G. L., Sipe J. E. (1996). Pure Appl. Opt..

[cit43] Alvarez M., Khoury J., Schaaff T., Shafigullin M., Vezmar I., Whetten R. (1997). J. Phys. Chem. B.

[cit44] Palpant B., Prevel B., Lerme J., Cottancin E., Pellarin M., Treilleux M., Perez A., Vialle J. L., Broyer M. (1998). Phys. Rev. B: Condens. Matter Mater. Phys..

[cit45] De G., Tapfer L., Catalano M., Battaglin G., Caccavale F., Gonella F., Mazzoldi P., Haglund Jr R. F. (1996). Appl. Phys. Lett..

[cit46] Aryal S., Remant B., Dharmaraj N., Bhattarai N., Kim C., Kim H. (2006). Spectrochim. Acta, Part A.

[cit47] Kumar S., Rai S. B. (2010). Indian J. Pure Appl. Phys..

[cit48] Guo Y., Yan H. (2008). J. Carbohydr. Chem..

[cit49] Hvoslef J., Kleboe P. (1971). Acta Chem. Scand..

[cit50] Socrates G. , Infrared and Raman Characteristic Group Frequencies: Tables and Charts, Wiley, U.K., 3rd edn, 2004

[cit51] Singh P., Singh N., Yadav R. A. (2010). J. Chem. Pharm. Res..

[cit52] Xiong J., Wang Y., Xue Q., Wu X. (2011). Green Chem..

[cit53] Berga R. (2015). Appl. Spectrosc. Rev..

[cit54] Hong H., Park C., Gong M. (2010). Bull. Korean Chem. Soc..

[cit55] Borchert H., Shevchenko E. V., Robert A., Mekis I., Kornowski A., Grubel G., Weller H. (2005). Langmuir.

[cit56] Ganeev R. A., Ryasnyansky A. I. (2006). Appl. Phys. B.

[cit57] Pollnau M., Hardmann P. J., Kern M. A., Clarkson W. A., Hanna D. C. (1998). Phys. Rev. B: Condens. Matter Mater. Phys..

[cit58] Dolgaleva K., Boyd R. W. (2012). Adv. Opt. Photonics.

[cit59] Gómez L. A., De Araújo C. B., Brito Silva A. M., Galembeck A. (2007). J. Opt. Soc. Am. B.

[cit60] He T., Wang C., Pan X., Wang Y. (2009). Phys. Lett. A.

[cit61] shahriari E., Yunus W. M. M., Naghavi K., Talib Z. A. (2010). Opt. Commun..

[cit62] Ganeev R. A. (2005). J. Opt. A: Pure Appl. Opt..

[cit63] Wang J., Blau W. J. (2009). J. Opt. A: Pure Appl. Opt..

[cit64] Ryasnyanskiy A. I., Palpant B., Debrus S., Pal U., Stepanov A. (2007). J. Lumin..

[cit65] Frare M. C., Signorini R., Weber V., Bozio R. (2015). Proc. SPIE.

[cit66] Henari F. Z., Dakhel A. A. (2008). J. Appl. Phys..

[cit67] Sarkhosh L., Aleali H., Karimzadeh R., Mansour N. (2010). Phys. Status Solidi A.

